# Clinical feasibility of targeted cardiac resynchronization lead delivery using a 3D MRI cardiac model

**DOI:** 10.1186/1532-429X-14-S1-P217

**Published:** 2012-02-01

**Authors:** John Stirrat, Raymond Yee, Andrew Krahn, Lorne J Gula, Peter Leong-Sit, George J Klein, David Scholl, Aashish Goela, Maria Drangova, James A White

**Affiliations:** 1 Imaging, Robarts Research Institute, London, ON, Canada; 2 Medicine, Division of Cardiology, London Health Sciences Centre, London, ON, Canada; 3 Radiology, London Health Sciences Centre, London, ON, Canada; 4 Medical Biophoysics, University of Western Ontario, London, ON, Canada

## Background

Cardiac resynchronization therapy (CRT) aims to reduce dyssynchronous contraction through simultaneous pacing of the right ventricular (RV) septum and left ventricular (LV) lateral wall. Up to 40% of patients do not respond, largely attributed to lack of dysynchrony and/or transmural scar at pacing sites. In this pilot study we tested the feasibility of guiding LV and RV leads to “optimal” segmental targets using a MRI-based 3D surface rendered cardiac model.

## Methods

**Figure 1:**
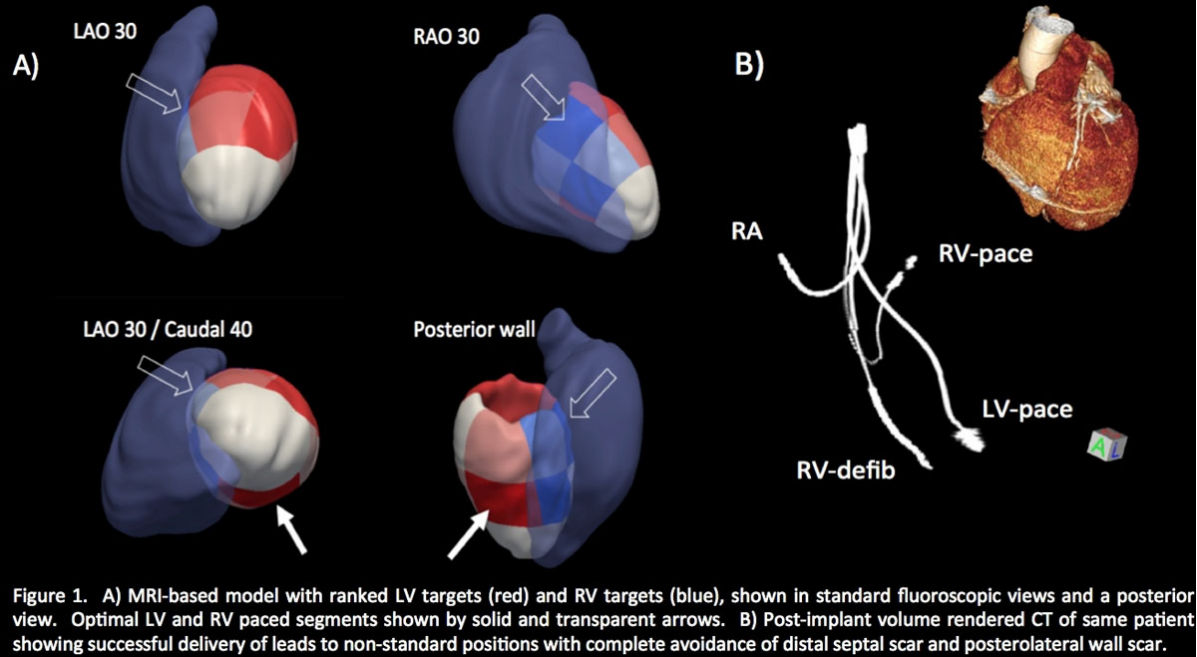


Ten consecutive patients planned for CRT were recruited. All patients underwent cardiac MRI inclusive of cine and delayed enhancement (DE) imaging using a 3T scanner. A blinded interpreter determined the time to maximal radial wall thickening (TmWT) and myocardial scar burden for each of 16 segments. All potential LV lead targets were ranked according to scar burden (lowest first) and then sub-ranked by TmWT (highest first). All potential RV lead targets were ranked according to scar burden (lowest first). These rankings were encoded onto a surface rendered cardiac model, displayed in standard fluoroscopic views (Figure 1A) and used to direct lead placement by fluoroscopy. A cardiac gated CT was then performed at a 1-month follow-up visit (Figure 1B) to assess procedural success for target achievement.

## Results

Mean age and LVEF was 68.5 ±6.7 years and 25.6 ±10.8%, with a mean NYHA class of 2.9 ± 0.6. “Optimal” LV and RV pacing sites differed from conventional pacing sites (basal posterolateral segment and RV apex) in 9 and 4 patients, respectively. All patients successfully underwent CRT device implantation with a mean fluoroscopy time of 23 ± 10.9 minutes. Post-device CT imaging showed LV and RV leads to be successfully delivered to the “optimal” or immediately adjacent segment in 90% and 90% of patients, respectively. The only documented complication was a small pericardial effusion not requiring drainage in a patient that was prescribed conventional lead positioning by the model.

## Conclusions

In this pilot study we demonstrate that a targeted approach to CRT lead placement using an MRI-based cardiac model is clinically feasible. A prospective clinical trial evaluating the clinical benefit of this approach is planned.

## Funding

J.A.W is a clinician scientist with the Heart and Stroke Foundation of Ontario, Canada. This research was supported by the Heart and Stroke Foundation grant # NA6488 (PI: J.A.W.) and by the Canada Foundation of Innovation (CFI) Leaders Opportunity Fund.

